# Supplementary material for: Impact of the COVID-19 Pandemic on Influenza Vaccination Coverage of Healthcare Personnel in Alicante, Spain
Source: Vaccines (Basel). 2024 Apr 1;12(4):370. doi: 10.3390/vaccines12040370 (PMC11055171; doi:10.3390/vaccines12040370)
Supplement: Supplementary file 1 [file vaccines-12-00370-s001.zip › vaccines-2921836-supplementary.pdf]

DATA COLLECTION FORM: Reasons to get vaccined

- Registration number (NR):

DATE OF BIRTH: __________

GENDER: 1. Male 2. Female

PROFESSIONAL CATEGORY (CAT):

1. Physician 2. Nurse 3. Assistant Nursing/technicians 4. Others

- VACCINATED (VAC): 1.YES. 2.NO

- VACCINATION DATE (FVAC): _____ /_____ /_____

- REASONS TO GET VACCINED (MOT):
    1. Protect my health
    2. It is my obligation
    3. I think it is advisable
    4. It's free
    5. I was vaccinated previously
    6. I suffered flu in other years
    7. Protect the health of my family
    8. Protect the health of patient
    9. Doctor's recommendation
    10. I suffer a chronic disease
    11. I live with people >65 years old.
